# Vulnerability of China’s nearshore ecosystems under intensive mariculture development

**DOI:** 10.1007/s11356-015-5239-3

**Published:** 2015-09-02

**Authors:** Hui Liu, Jilan Su

**Affiliations:** 1 Yellow Sea Fisheries Research Institute, Chinese Academy of Fishery Sciences, Qingdao, China; 2 Second Institute of Oceanography, State Oceanic Administration, Hangzhou, China

**Keywords:** Large-scale intensive mariculture, Impact on marine ecosystems, Sustainable development, Ecosystem approach to aquaculture

## Abstract

Rapid economic development and increasing population in China have exerted tremendous pressures on the coastal ecosystems. In addition to land-based pollutants and reclamation, fast expansion of large-scale intensive mariculture activities has also brought about additional effects. So far, the ecological impact of rapid mariculture development and its large-scale operations has not drawn enough attention. In this paper, the rapid development of mariculture in China is reviewed, China’s effort in the application of ecological mariculture is examined, and the vulnerability of marine ecosystem to mariculture impact is evaluated through a number of examples. Removal or reduced large and forage fish, due to both habitat loss to reclamation/mariculture and overfishing for food or fishmeal, may have far-reaching effects on the coastal and shelf ecosystems in the long run. Large-scale intensive mariculture operations carry with them undesirable biological and biochemical characteristics, which may have consequences on natural ecosystems beyond normally perceived spatial and temporal boundaries. As our understanding of possible impacts of large-scale intensive mariculture is lagging far behind its development, much research is urgently needed.

## Introduction

Since the early 1980s, China’s economy has developed rapidly with an average annual GDP growth rate over 9 %, according to its National Bureau of Statistics. The growth rate of the 11 provinces and metropolises along the coasts exceeded 10 %. In fact, with only 13 % of China’s land area, in 2011, the 11 coastal provinces and metropolises housed about 42 % of its population and produced over 57 % of the national GDP (CCICED Task Force 2013).

Rapid economic growth and urbanization have brought about tremendous pressure on the coastal and marine ecosystems, resulting in the loss or deterioration of many of their services. Land-based pollutant and coastal reclamation are the two most detrimental factors to the health of the ecosystems (CCICED Task Force 2013). According to the official bulletin, in 2009, over half of China’s nearshore waters were classified as below the water quality standard and about 20 % as severely below standard. Occurrence of recorded events of harmful algal blooms (HABs) rose sharply to about 80 yearly, covering a total area exceeding 15,000 km^2^ (op cit). Between 1990 and 2008, the average reclamation area in China reached 285 km^2^ yearly and a further 5780 km^2^ was planned for over the next 10 years: The fast pace of reclamation has caused significant coastal landscape fragmentation and loss of biodiversity, as well as destruction of habitats for fish and loss of feeding grounds for shorebirds, leading to sharp decline in fisheries resources and bird species (Wang et al. 2014). In addition, overfishing, rapid expansion of mariculture, and construction of large number of hydraulic works on land all had compounded the impacts on China’s coastal and marine ecosystems (CCICED Task Force 2013).

Spurred by both improved culture technology and rise in domestic demand for high-quality seafood, there has been a revolutionary growth of mariculture in China over the last 30 years. China is credited with 63.3 and 54 %, respectively, of the world’s farmed marine food fish and aquatic plant production (FAO 2014), which are usually farmed in large-scale aquaculture operations. For such high production, extensive patches of tidal flats and nearshore waters have been converted for mariculture use. However, so far, the ecological impact of rapid mariculture development and its large-scale operations has not drawn sufficient attention in China. Attention is usually directed at the food provision function of mariculture, while the interference of mariculture with other ecosystem services has not been fully recognized.

In this paper, the rapid development of mariculture in China is reviewed, China’s effort in the implementation of ecological mariculture practices is examined, and the vulnerability of marine ecosystems to impacts of mariculture is evaluated, pertaining to large-scale operations. Considering the current scale of mariculture worldwide and its significant upward trend, impacts of the world’s intensive mariculture on the ocean ecosystems are far from negligible. Understanding the true state of impact of intensive mariculture on coastal and marine ecosystems will facilitate consideration of precautionary measures to ensure sustainable growth of world mariculture.

## Mariculture in China

**Fig. 1:**
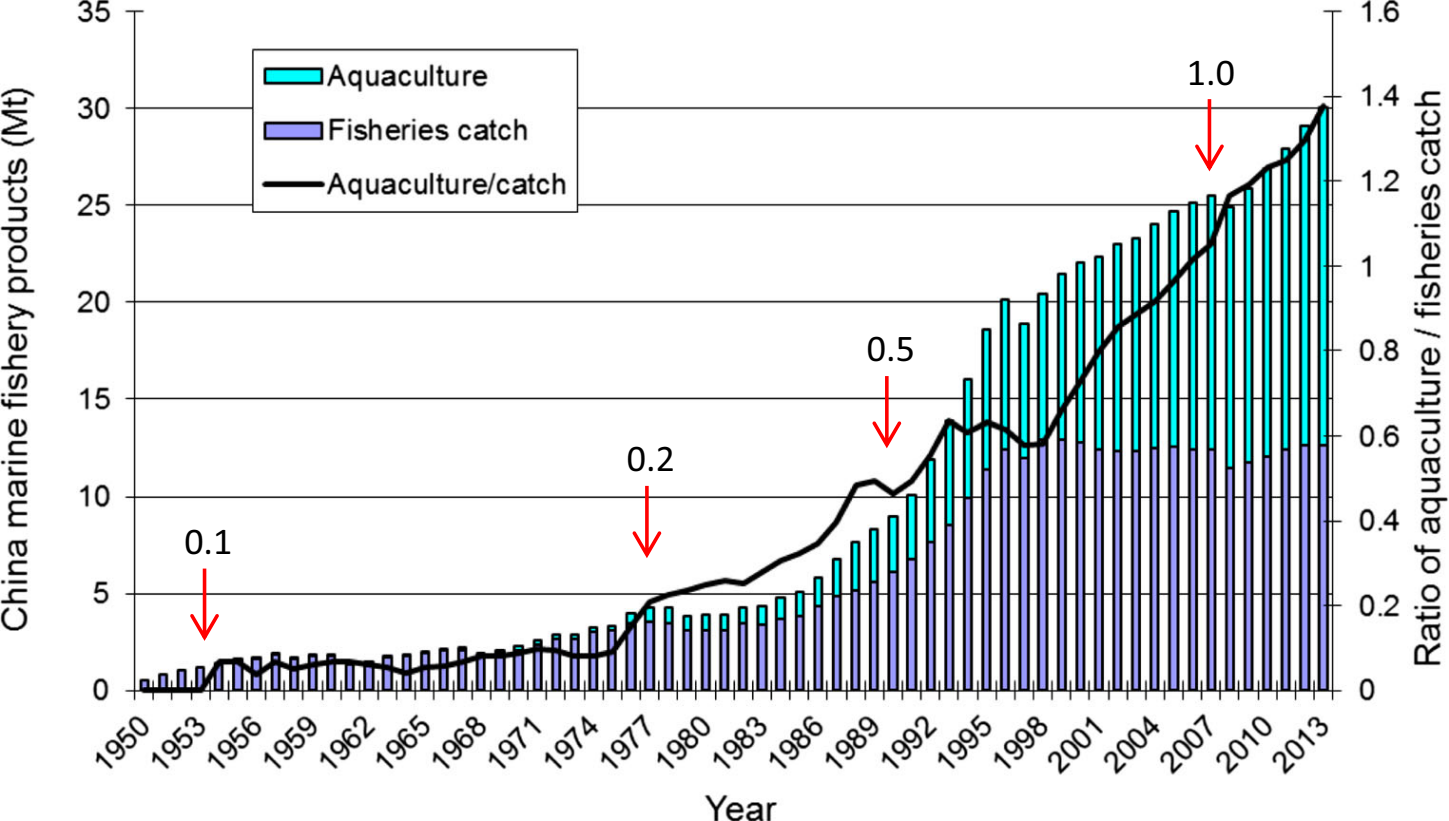
Growth of mariculture production and fishery catch during 1950–2013. The *number above each red arrow* denotes the ratio of “aquaculture/fishery catch” for that year (data source: MOA 1979–2014)

Along with the world’s rapid growth in mariculture over the recent decades, China’s mariculture has also undergone unprecedented development (Fig. 1). Since the middle 1970s, both the mariculture production and capture fisheries experienced fast growth. However, after the middle 1990s, the capture fisheries became stagnant while mariculture kept on expanding. The ratio between the mariculture production and fishery catch was almost nil in early 1950s, reached 20 % in 1975, 50 % in 1988, and 100 % in 2006, and stood at 140 % in 2013. This is in sharp contrast to the ratio for Europe (18 %) and for other continents (<15 %) (data source: FAO FishStatJ). In 2012 China’s mariculture contributed to 54 and 63.3 %, respectively, of the world’s farmed aquatic plants (largely seaweeds) and marine food fish production (FAO 2014). In light of the depletion of wild fish stocks and the continuing rise in domestic seafood demand, mariculture will undoubtedly lead the future growth of Chinese aquaculture in the long run.

**Fig. 2:**
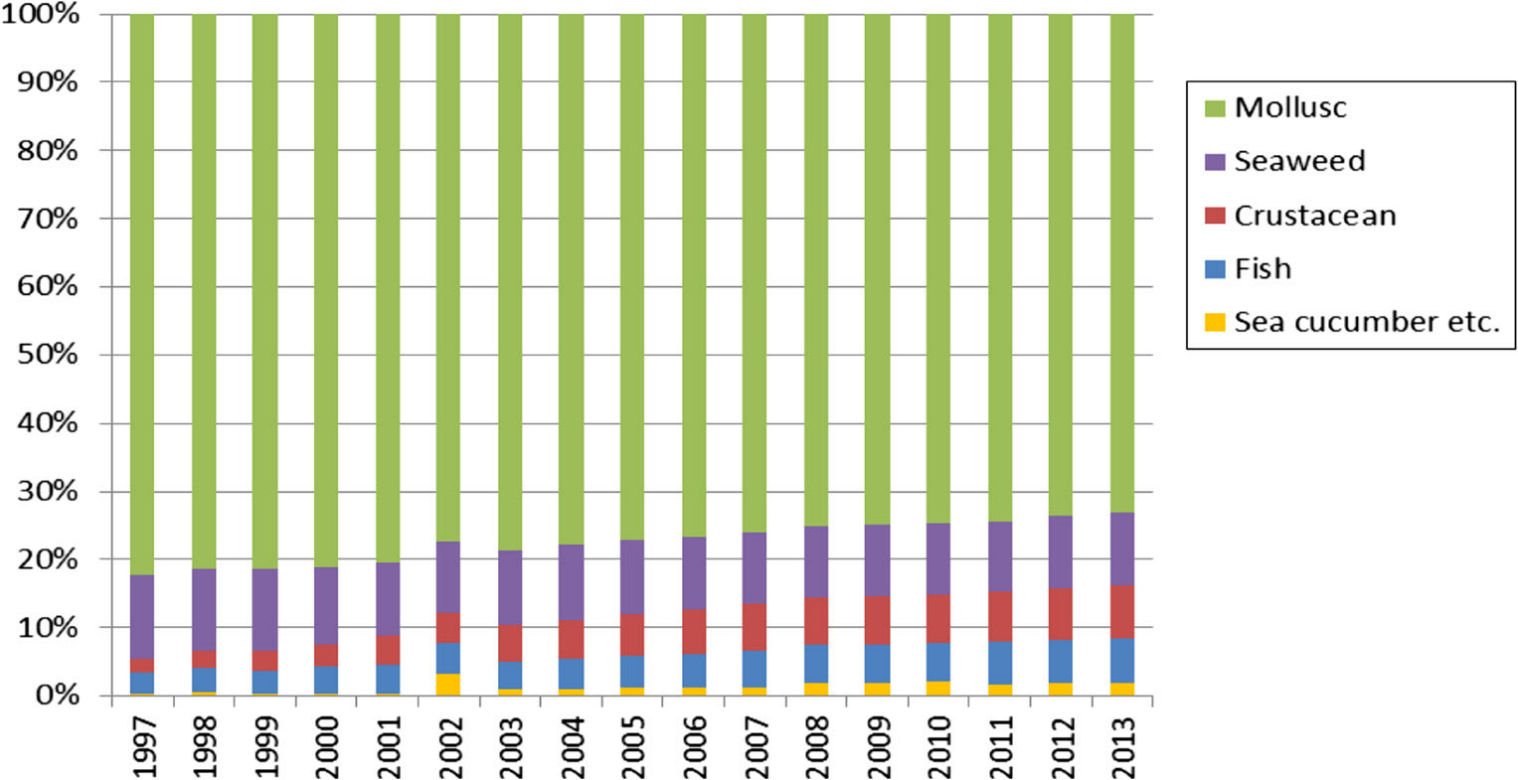
Change of the composition of China’s mariculture over time (data source: MOA 1998–2014)

Throughout the 1950∼1980, mariculture species in China were largely confined to seaweeds and bivalves, which are extractive species relying on natural supply of nutrients and plankton. Monoculture on longlines over tidal flats was the main type of operation. Commercial culturing of shrimp, finfish, and other high-value species began in the early 1980s, when land-based hatcheries, earthen ponds, and net cages were rapidly set up. The trend of “fishing down and farming up the food web” has been significant in the world fishery industry for decades (Naylor et al. 2000), and China is no exception. In the last 15 years, mariculture production of fed species (finfish and crustaceans) increased sharply from <5 to >15 %, showing a clear upward trend into the future (Fig. 2). This reflects the increased domestic market demand for high-value seafood, corresponding to the economic growth in China. At the same time, this will no doubt bring about increased wastes from mariculture, because, unlike the freshwater finfish which are mostly planktonic filter feeders or omnivorous, marine finfish and crustaceans are generally carnivorous and high in trophic level. Paying attention to this trend is important for rational planning and ecosystem-based management of mariculture.

**Fig. 3:**
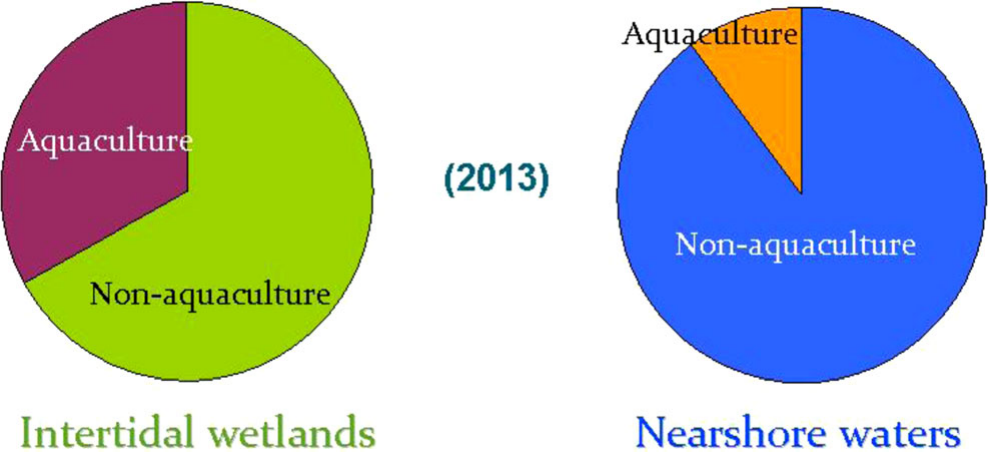
Portion of area used for mariculture in, respectively, intertidal wetlands and nearshore waters of China in 2013. Calculation based on coastal wetland area data from the SFA (2011) and aquaculture area data from MOA (2014)

Along with the increase of production, large portions of area from both the intertidal wetlands and nearshore waters of China were used for mariculture (Fig. 3). According to the Chinese fishery statistics (MOA 2014), the total mariculture acreage amounted to 2.32 Mha in 2013, among which 0.8 Mha was converted from intertidal wetlands (nearly one third of China’s total) and 0.9 Mha from the nearshore waters (nearly 10 % of China’s total) was used for mariculture purpose.

## China’s approach to ecological mariculture

Aquaculture is basically an economic activity that consumes resources, impacts on biodiversity in different levels and ways, and discharges wastes, hence affecting the environment (e.g., Naylor et al. 2000). With the worldwide rapid development of aquaculture since the 1970s, especially in developing countries, there was first a focus on productivity and benefits, leading to a general scaling up and overstocking. The resulting aquaculture epidemics and mass mortalities raised awareness of ecological carrying capacity of the aquaculture systems (e.g., Grant et al. 2007). This concept soon evolved into ecological aquaculture, with ecosystem approach to aquaculture (EAA) as its implementation strategy (Soto et al. 2008).

Conceptually, EAA embodies the principle of ecosystem-based management by promoting conservation and sustainable use of resources. In implementing EAA, it is necessary to define the ecosystem boundaries in both space and time. For this, three levels of scale have been identified, namely, the farm, the watershed/aquaculture zone, and the global (Soto et al. 2008). In practice, boundaries for the second level, i.e., the watershed/aquaculture zone level, sometimes are not easy to delineate, both managerially and ecologically. For a fjord-like inlet where the physical boundaries are more or less defined, management tools including analysis models for evaluating aquaculture in an ecosystems approach were developed and proved to be useful (Nunes et al. 2011). However, because of the lack of a good understanding of the interaction between a mariculture operation and the marine ecosystems at large, presently, there are no agreed-on guidelines or practical measures for implementing EAA at the level of the watershed/aquaculture zone.

One innovative approach within EAA is the so-called integrated multi-trophic aquaculture (IMTA), in which multi-trophic species with complimentary trophic functions are cultured together. Traditional IMTA has been practiced in the world for thousands of years, including ancient China (Costa-Pierce 2010). Modern polyculture methods in China, as one of the eight techniques embodying China’s freshwater aquaculture, were reviewed for the first time in 1959 (CoCFAE 1961).

With the development of intensive aquaculture worldwide since the 1970s, monoculture of omnivorous or carnivorous marine species at high densities, including finfish and crustacean species, was initially carried out in China. The production boom in late 1980s and early 1990s brought about environmental pollution and disease outbreak among the stocks, leading to a crash in harvest. At the same time, technology improvement allowed for restructuring of the existing culture systems. There was then a clear transition in China from intensive monoculture to polyculture. The first successful attempt in marine polyculture was conducted in 1975, when kelp *Laminaria japonica* and mussels *Mytilus edulis* were co-cultured (Xie 1981; Dong 2011). Soon, polyculture in China evolved such that fed (fish and shrimp) and non-fed species (bivalves and seaweeds) were cultured together, where the non-fed species served as a means to recycle wastes and reduce pollution (e.g., Wu et al. 1980; Zhu 1981). Thus, the ecosystem perspective in traditional mulberry-fish pond system from ancient China regained appreciation. Such IMTA mode ecological aquaculture is now commercially practiced in China (Fang 1996; Dong 2011).

A major IMTA demonstration site in China is Sanggou Bay, at the tip of Shandong Peninsula in eastern China, where, according to local statistics, seaweed, shellfish, and finfish are cultured at a ratio of approximately 7:3:1 with an annual seaweed production of about 200 thousand tons (Zhang XJ, Head of Ocean and Fishery Bureau of Rongcheng, private communication). Based on both research findings and technology improvement, the ratio and layout of the seaweed longlines, shellfish lantern nets, and finfish net cages were constantly optimized to minimize the environmental impact from this major IMTA system (Tang et al. 2013, Shi et al. 2013). Although IMTA has become an important component for mariculture in China, there is no official statistics on its scale and total production because it is not yet classified as one standard aquaculture mode.

At the same time, IMTA has been met with worldwide enthusiasm during the last decade, as an effective way to deal with the wastes and improve the output of aquaculture (Barrington et al. 2009; Chopin 2012). In the present practice of IMTA, there are various approaches to achieve the basic goals that feed and wastes are recycled within the system. With different species composition and spatial configuration, IMTA has been applied to both pond and open-water mariculture systems. Although there were attempts to study the nutrient balance within an IMTA system from a modeling point of view (e.g., the study of ratios between fed and extractive species in IMTA farms (Reid et al. 2013)), lack of thorough understanding of environmental impact of IMTA remains one major concern (e.g., IMTA White Paper 2010).

From the point of view of EAA, boundary delineation of an IMTA system at the watershed/aquaculture zone level is often not straightforward. This results in rather complicated interaction between the culture system and the natural habitats. For example, large-scale IMTA in shallow bay will significantly alter the tidal currents, resulting in a reduced flushing capability of the bay, as well as insufficient nutrient supply to the bay from the offshore water (Shi et al. 2011). In addition, organic wastes do accumulate in the sediments of IMTA area and alter both the benthic and water column chemical parameters (Ren et al. 2014). Given the physical conditions of any particular IMTA site, it is extremely difficult, if not impossible, to recycle all the nutrients and wastes within an IMTA system.

## Vulnerability of the nearshore ecosystem

As commented in the “Introduction” section, over the last 30 years, the nearshore ecosystems of China have been under multiple pressures of human activities, including land-based pollutants, reclamation, port construction, overfishing, and mariculture. Early concern about the impact from mariculture was limited to its waste discharge pollution and escapees. However, as China’s mariculture rapidly expands, there are evidences now that large-scale intensive mariculture may bring about undesirable ecological consequence far beyond the normally perceived boundaries of its operation. The combined impact from mariculture and other human activities has an amplification effect that significantly increases the vulnerability of China’s coastal and marine ecosystems. In the following, we will use a few examples to illustrate this point.

### Degradation and destruction of natural habitats

As is well known, intertidal wetlands and nearshore waters are critical habitats for many marine organisms, including both large and forage fish. As extensive tidal flats and wetlands are transformed to aquaculture ponds, the natural ebb and flow of tides disappear, along with the local benthic organisms and migrating shore birds (Yang et al. 2004, Chen and Ouyang 2014). Mariculture not only introduces pollutants into nearshore material cycle, but also interferes with local hydrologic processes which can disrupt the success of recruitment in the early life of fisheries (e.g., Dong et al. 2007). In open-water aquaculture, the environmental impact is heavily influenced by local circulation (Han and Liu 2014). It is noteworthy that mariculture impact on a sheltered bay is usually more significant due to its limited environmental carrying capacity. Large-scale net cages and longline facilities in shallow bays significantly slow down the tidal currents, resulting in weakened flushing capability of the bay, reduced nutrient supply to the inner bay, increased deposition, and reduced productivity of phytoplankton (Ji et al. 1998; Liu et al. 2004; Shi et al. 2011). In addition, accumulation of waste deposits under shellfish longlines or finfish net cages may severely affect the macro-benthos and change their biodiversity, composition, and distribution (Gao et al. 2003; Yang et al. 2007; Wang et al. 2011). In the long run, the establishment of a mariculture system may result in fragmentation of the original natural ecosystems and disappearance or significant change in some of the original biotic communities (Liu and Fang 2014).

Furthermore, mariculture also imposes deleterious effects on the natural fauna and flora through its chemical or pharmaceutical discharge. To eliminate harmful or undesirable organisms in many of the extensive pond mariculture, pellouxite and other disinfectants have been used in high concentrations before or during the culture period (Luo 2012). When the treated water is discharged without further treatment, these chemicals will invariably cause high mortality to the nearby wild fauna and flora.

Although such impacts are well known, in the case of China, the magnitude of these impacts on the nearshore ecosystems needs to be assessed against the scope of the human activities. As discussed in the “Introduction” section, natural wetlands in China suffered great loss and degradation over decades. More than 50 % of the coastal wetlands were lost due to reclamation for various uses, including mariculture (An et al. 2007). The loss of associated ecosystem services due to reduced purification capacity, failure in recruitment of fishery stock, and reduced biodiversity was estimated to amount to 46 billion USD per year (op cit).

**Fig. 4:**
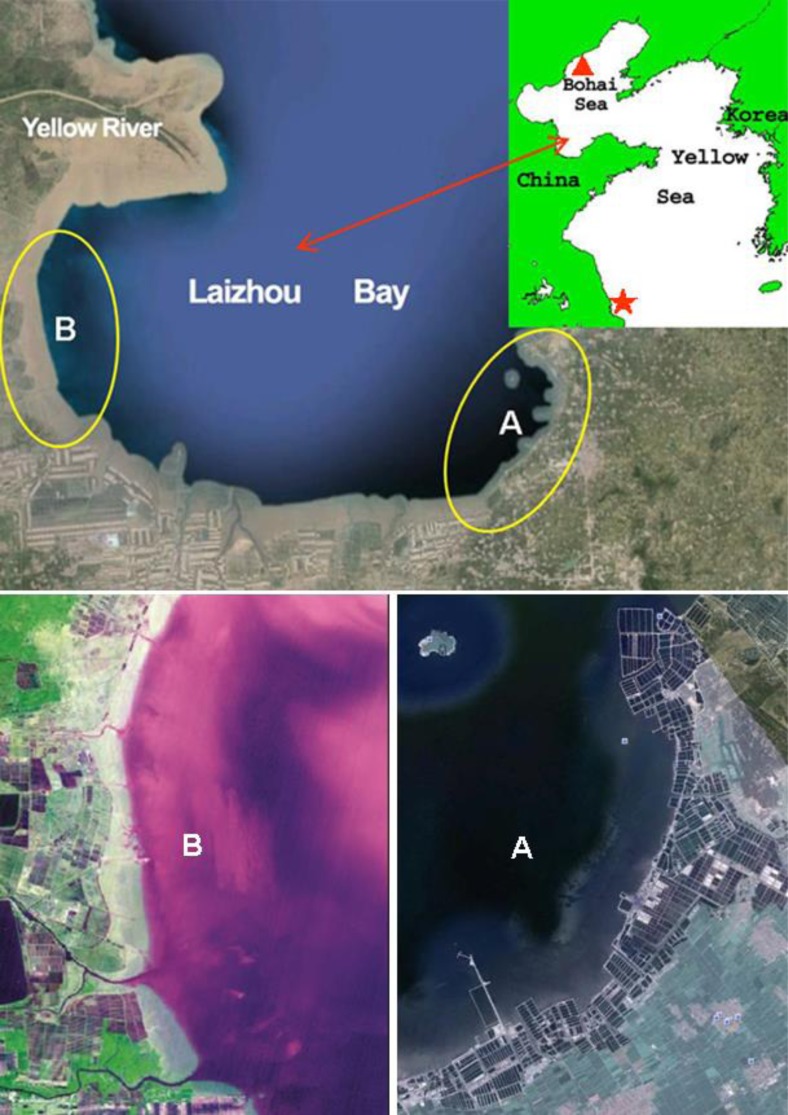
Extensive mariculture facilities along the shores of the Laizhou Bay. In the inserted map on the *top figure*, the *red triangle* and *star* denote, respectively, the location of the large-scale bivalve culture and the extensive Su-Bei tidal shoals

For example, the Laizhou Bay of Bohai Sea has always been used as spawning grounds for many fishery species of the Bohai Sea and Yellow Sea (Deng and Jin 2000; Xu and Chen 2010). However, presently, most of the estuaries and wetlands around the bay are severely affected by mariculture activities (Fig. 4). Furthermore, not shown in the figure, there are also large areas of longline mariculture over the shallow waters in the bay. It is possible that most of the nursery grounds for the fisheries are either lost or severely degraded. Thus, in addition to overfishing, reclamation and mariculture activities may also contribute to the collapse of regional fishery (Zuo and Lin 2008; Wang et al. 2014).

The loss of coastal wetlands along with its biodiversity is not unique in China. As a matter of fact, there has been extensive loss of tidal flats around the whole Yellow Sea region, associated with economic development in China and the two Koreas. It was found that 28 % of the Yellow Sea tidal flats existing in the 1980s had been reclaimed by the late 2000s (Murray et al. 2014).

### Pressure on wild fisheries

There have been very few observations on the direct aquaculture impact on wild fishery, especially regarding a particular species and over a significant length of time. According to a 7-year study in Canada (Loucks et al. 2014), net cage finfish aquaculture produced odors and changed benthic habitat and water quality, which may affect the behavior and movement of wild fishery species (e.g., lobster). It was found that, within 2 years of a finfish aquaculture operation, ovigerous lobsters abandoned the area and overall lobster abundance decreased; the female lobsters returned to this area only when fish farming was discontinued. On the other hand, aquaculture may provide food for wild species. For example, aggregations of wild fish communities were repeatedly observed in the vicinity of aquaculture farms in the Mediterranean (Grigorakis and Rigos 2011).

In addition to habitats, marine capture fisheries and mariculture also compete for other ecosystem services, such as nutrients and feed, broodstock, and larval supply (Naylor et al. 2000). In China, the catch of wild broodstock for hatchery (such as Chinese shrimp *Fenneropenaeus orientalis*) and of young fish for grow-out or fattening (such as sea bass *Lateolabrax maculatus* and freshwater eel *Anguilla japonica*) has been going on for many years (Niu 1982; Gao and Zhang 2002; Zhi et al. 2013). The above-mentioned human activities together imposed devastating effect on the wild stock and also defeated large-scale restocking efforts conducted in China, including artificial reefs and annual release of tens of billions of fishery seedlings (Shan et al. 2012).

**Fig. 5:**
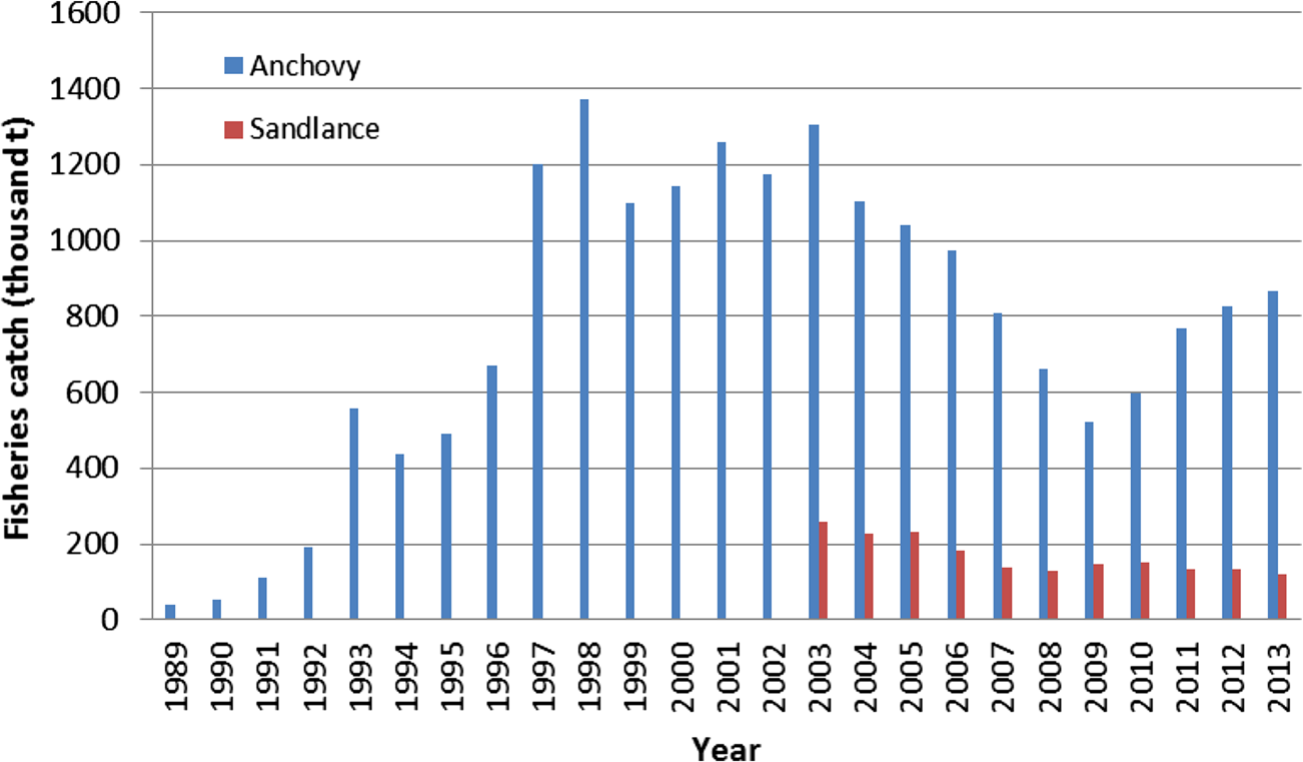
Fishery catch of anchovy and sandlance in China during 1989–2013 (data source: MOA (1990–2014))

Another impact of mariculture on wild fishery is the harvest of forage fish, which is either directly fed to aquaculture or processed into fishmeal. Generally speaking, in China, about one third of forage fish (about 4 Mt) are fed directly to aquaculture species (Mai 2010), although there is large site-specific variability. Annual production of these small forage fish amounted to several million tons, though not all such productions are recorded in the official fishery statistics (op cit). Statistics of specific forage fisheries varied widely over the decades, reflecting overfishing pressure on these species (Fig. 5).

Jackson et al. (2001) have shown that overfishing of large fish fundamentally altered coastal marine ecosystems during each of the cultural periods examined. Forage fish is also an important part of the regional marine ecosystems. With pressure from both habitat loss/degradation and overfishing, it is hard to imagine how the regional coastal and nearshore ecosystems will evolve when both the large and forage fisheries are destroyed.

### Proliferation of opportunistic species

**Fig. 6:**
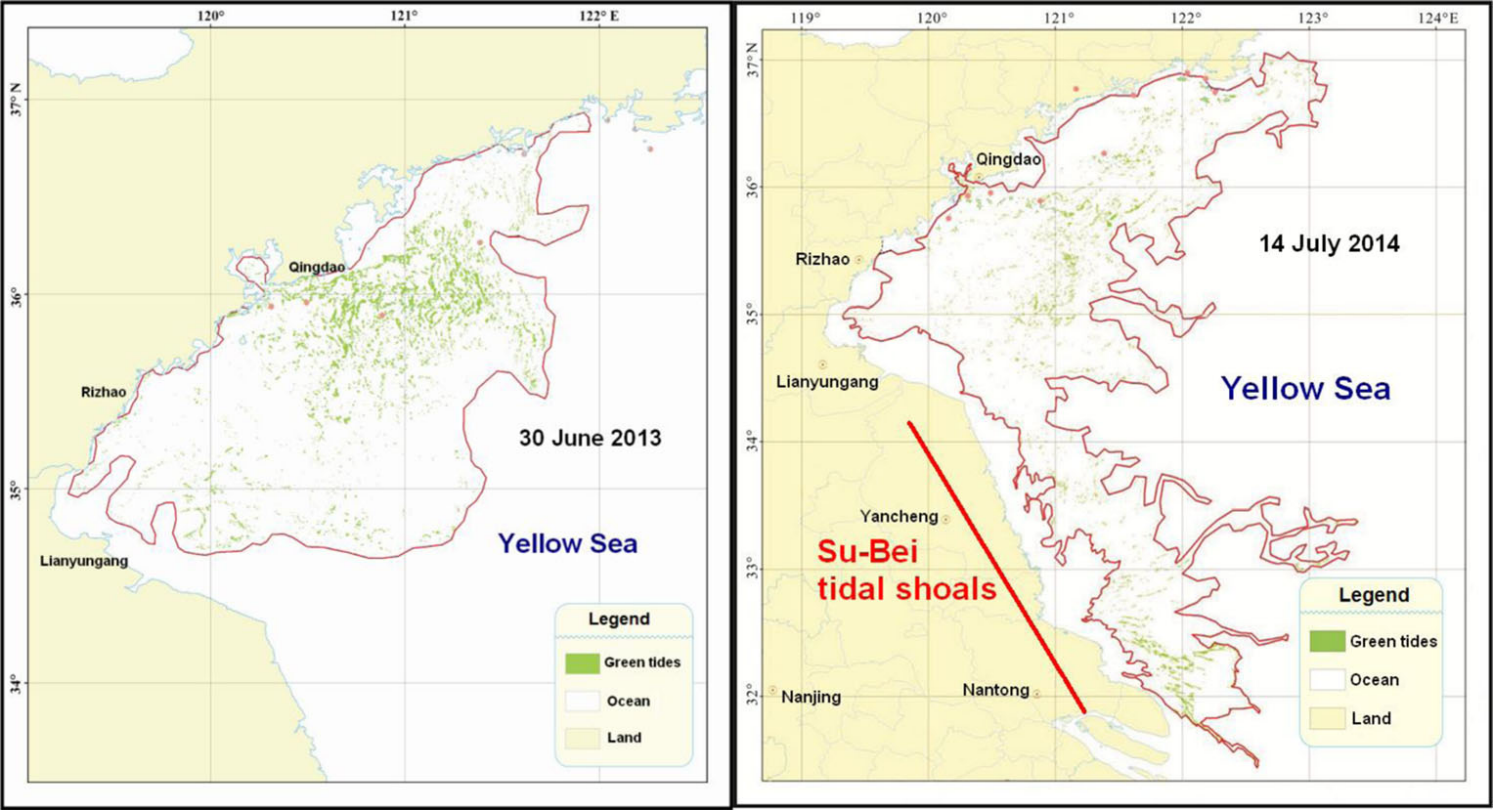
Distribution pattern of the green tides over Yellow Sea on 30 June 2013 and 14 July 2014 (figure source: SOA 2014, 2015)

Since 2007, there has been yearly outbreak of large-scale green tides over the Yellow Sea in summer. In 2013, at its peak, it covered a total area 790 km^2^ distributed over a wide area of 29,700 km^2^, while, in 2014, the numbers were, respectively, 540 and 50,000 km^2^ (SOA 2014, 2015). Figure 6 shows the distribution pattern of the green tides on 30 June 2013 and 14 July 2014. The green tides are caused by *Ulva prolifera* (*Enteromorpha prolifera*), a macro-algae species often found along the coasts of temperate Asia, including China. Proliferation of *U. prolifera* is associated with the large-scale mariculture system of *Porphyra yezoensis* over the extensive Su-Bei tidal shoals of China (Figs. 4 and 6) off the west boundary of the Yellow Sea (Liu et al. 2009).

Micropropagules of *U. prolifera* generally exist in the nearshore water of Yellow Sea. The micropropagule will grow only after attaching itself to a periodically exposed surface, including the rafts used for the *P. yezoensis* culture. A variety of macro-algae species would attach and grow on these rafts from late autumn, including *U. prolifera*, *Ulva linza*, *Ulva compressa*, *Ulva intestinalis*, and *Ulva clathrata. U. prolifera* became one of the most dominant species only in late March to mid-May (Fan et al. 2015). At the harvest of *P. yezoensis* in April, the attached macro-algae were stripped off and left on the shoals. Most of the macro-algae left on the shoals were drowned except *U. prolifera* which can float during photosynthesis. Once leaving the shoals, the floating *U. prolifera* would drift in an N-NE direction driven by the prevailing winds. The floating *U. prolifera* grew at an initial daily rate of 20–30 % and soon evolved to green tides (op cit). Some of the drifting green tides landed on the shores of Shangdong Province in June/July while the rest eventually sank offshore.

**Fig. 7:**
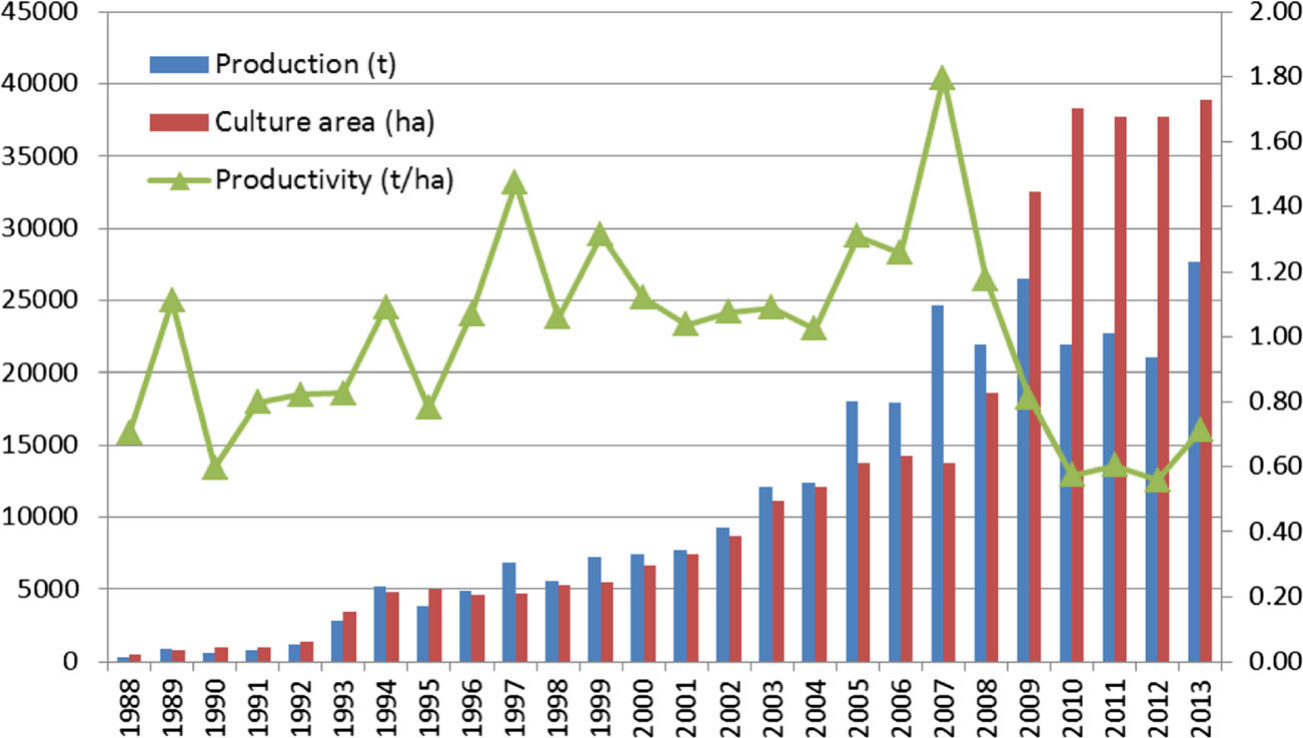
Yearly *P. yezoensis* culture production, culture area, and productivity in Jiangsu Province in 1988–2013 (source of data: MOA 1989–2014)

Figure 7 gives the yearly production, culture area, and productivity of the *P. yezoensis* culture in Jiangsu Province between 1988 and 2013 (most of this culture is over the Su-Bei Shoals). It is noted that the yearly production of *P. yezoensis* reached a plateau after 2007 even though the cultured area kept increasing. The productivity underwent a sharp drop then. The year 2007 was when the green tides over the Yellow Sea first became noticeable. From 2008 to the present, there is a yearly outbreak of green tides covering 250–2100 km^2^ distributed over 20,000–60,000 km^2^. It is possible that certain bloom-state threshold for the opportunistic species *U. prolifera* was reached after 2007, likely related to the size of the culture area of *P. yezoensis* (Fig. 7).

There is no reported estimate on the portion of the floating mass of *U. prolifera* that sink offshore, nor is there any study on the fate of these submerged macro-algae. It is well known that, in summer, the Yellow Sea Cold Water (YSCW), a water mass, occupies the central part of Yellow Sea deeper than 40 m below a sharp thermocline (e.g., Su 1998). Figures 7-8 and 7-6 in Hong (2012), respectively, showed that the bottom dissolved oxygen in YSCW is highest in spring and lowest in autumn, and in addition, over the past 30 years, it has a continuous decreasing trend with an average drop of about 0.77 mg l^−1^. A recent ocean acidification survey found that in late autumn of 2012, large area of YSCW had a saturation state of aragonite below 1.0 (SOA 2013), indicating a corrosive environment for many marine organisms (Fabry et al. 2008). It is known that YSCW serves as important oversummer grounds for *Calanus sinicus*, an important copepod providing a wide size spectrum of food for fish stocks (Wang et al. 2003). How the annual outbreak of *U. prolifera* blooms will exert further pressure on the ecosystem services of YSCW is yet to be studied.

### Alteration of biochemistry in water/sediment

Although mariculture ponds and enclosures and nearshore longline culture areas are usually counted as artificial wetlands, they are fundamentally different from natural coastal wetlands because their ecosystem services are significantly different. Mariculture is also a major driver on changes in the biogeochemistry of its neighboring water and sediment.

The deleterious environmental effects of the intensive and industrialized mariculture have been well recognized (e.g., National Research Council 2010). Finfish cage culture can cause significant rise in dissolved nitrogen in water column, particulate phosphorous in sediment, and chemical oxygen demand (COD) in both the water column and sediment (e.g., Holby and Hall 1991; Wang 1998). Large-scale oyster (*Crassostrea gigas*) culture may alter the sediment geochemistry by raising NH_4_^+^ and HPO_4_^2−^ by 7.7–11.5 and 1.8–8.0 times and sulfate reduction rates by 2.4–5.2 times (Hyun et al. 2013). Given the scale of bivalve culture in China (total annual production of 12.7 Mt over a total area of 1.6 Mha) (MOA 2014), the biochemical alteration of inshore sediments is considered significant enough to induce eutrophication, change in P/N ratio, and outbreaks of HABs (Bouwman et al. 2013). This was echoed by Hyun et al. (2013), who observed enhanced benthic microalgal biomass and primary production in the water column at the bivalve farms. As discussed in “China’s approach to ecological mariculture” section, it is difficult even for an IMTA system to recycle all the nutrients and wastes within its system. Indeed, eutrophication effect of polyculture (with finfish, shellfish, and seaweed) can be significant in a sheltered bay (Zhang et al. 2003).

Nutrients in the reduced form in the ocean are of particular concern. For example, reduced N forms were preferentially taken up during the harmful microalgae blooms of *Karenia mikimotoi* and *Prorocentrum donghaiense* in nearshore waters of the East China Sea (Li et al. 2009). Both of these are common HAB species in coastal China, and the blooms of *K. mikimotoi* usually cause great losses in mariculture (SOA 2005–2015).

Among all forms of mariculture pollutions, direct release of nutrients in the reduced form into the ocean is of more concern (Bouwman et al. 2013). Laboratory experiments showed that ammonia and urea excreted by the cultured animals were readily taken up by phytoplankton and could stimulate their growth (Arzul et al. 1996; Yang et al. 2004). When the level of such nutrient is higher than the assimilative capacity of the marine area, it may fuel the production of algae on which mixotrophic HABs may feed (op cit). In turn, such mixotrophic HABs can kill or intoxicate the mariculture organisms and increase risks to human health. The scallop *Argopecten irradians* culture at the nearshore region of the northwestern Bohai Sea (Fig. 4) is number one in China by area scale (Zheng et al. 2011). Coincidentally, large-scale *Aureococcus anophagefferens* brown tides, a form of mixotrophic HABs, have occurred in the northwestern Bohai Sea in early summer for consecutive years since 2009 and have caused significant negative impacts on the scallop culture there (Zhang et al. 2012). China is the third country in the world hit by brown tides, but at a much greater scale. At its peak, the brown tides covered an area of over 3000 km^2^ (op cit).

Particulate wastes from aquaculture usually accumulate in nearby sediments, driving an increase in COD value. As organic matter is decomposed by microbes through respiration, the redox potential will decrease, leading to bottom dissolved oxygen (DO) depletion (Wang 1998). Accumulation of wastes and microbial/phytoplankton biomass in proximity to mariculture sites may give rise to hypoxia conditions resulting in an anoxic layer of sediment and bottom waters (Yang et al. 2004), and this develops very often in stratified water. In fact, seasonal bottom oxygen depletion in western Bohai Sea, especially in the vicinity of large-scale bivalve culture sites, has now become normality and is getting worse (Zhai et al. 2012). The progressive depletion in the summer bottom dissolved oxygen in Bohai is rather alarming and raises further concern about Bohai Sea’s ecosystems. Observation showed that its minimum bottom oxygen level in August steadily dropped over the years, being 6.3 mg l^−1^ in 1982, 5.1 mg l^−1^ in 1992, 4.5 mg l^−1^ in 2006, 3.5 mg l^−1^ in 2011, and below 3 mg l^−1^ in 2014 (Zhai Weidong, private communication). Analysis by Zhai et al. (2012) indicated that both the bottom DO depletion and acidification in summer were caused by remineralization of biogenic particles, which were supplied by either coastal HABs or regional nearshore mariculture.

## Conclusion

As the world’s population keeps on increasing with high aspiration for better life, rapid intensive reclamation and large-scale mariculture practices will no doubt be replicated in developing countries around the world’s coasts. Such human activities are conducted over intertidal wetlands and nearshore waters, which lie in a narrow strip along the coast. These habitats are critical in the life history of many species of large and forage fish. At the same time, overfishing, either for food or for fishmeal, exerts tremendous pressure on the stock of both large and forage fish. During past cultural periods, overfishing of large fish was shown to have fundamentally altered coastal marine ecosystems (Jackson et al. 2001). If the large and forage fish are sharply reduced, both the coastal and shelf ecosystems will likely be significantly altered.

Mariculture replaces intertidal wetlands with artificial wetlands and brings artificial ecosystems into nearshore waters. These artificial ecosystems bring with them different but undesirable biological and biochemical characteristics. Spatial and temporal extents of the effects of these artificial ecosystems, i.e., the boundaries for the watershed/aquaculture zone level, are often hard to define. The speed of mariculture development far outpaces the progress of our knowledge about its impacts. Examples given above show that the coastal and nearshore ecosystems are rather vulnerable when facing intensive large-scale mariculture development.

The fast pace of the worldwide coastal development will likely cause the coastal and nearshore ecosystems to evolve into states unfriendly to either the coastal communities or the ocean ecosystems at large. As our understanding of such possible changes is lagging far behind the economic and social development, much research is urgently needed, including those related to large-scale intensive mariculture.
